# Emotional eating across different eating disorders and the role of body mass, restriction, and binge eating

**DOI:** 10.1002/eat.23477

**Published:** 2021-03-03

**Authors:** Julia Reichenberger, Rebekka Schnepper, Ann‐Kathrin Arend, Anna Richard, Ulrich Voderholzer, Silke Naab, Jens Blechert

**Affiliations:** ^1^ Department of Psychology, Centre for Cognitive Neuroscience Paris‐Lodron‐University of Salzburg Salzburg Austria; ^2^ Schoen Clinic Roseneck Prien am Chiemsee Germany; ^3^ Department of Psychiatry and Psychotherapy University Hospital of the LMU Munich Munich Germany; ^4^ Department of Psychiatry and Psychotherapy University Hospital Freiburg Freiburg Germany

**Keywords:** anorexia nervosa, binge‐eating disorder, bulimia nervosa, emotional eating, emotions, obesity, questionnaire

## Abstract

**Objective:**

Different subtypes of eating disorders (ED) show dysfunctional eating behaviors such as overeating and/or restriction in response to emotions. Yet, systematic comparisons of all major EDs on emotional eating patterns are lacking. Furthermore, emotional eating correlates with body mass index (BMI), which also differs between EDs and thus confounds this comparison.

**Method:**

Interview‐diagnosed female ED patients (*n* = 204) with restrictive (AN‐R) or binge‐purge anorexia nervosa (AN‐BP), bulimia nervosa (BN), or binge‐eating disorder (BED) completed a questionnaire assessing “negative emotional eating” (sadness, anger, anxiety) and “happiness eating.” ED groups were compared to BMI‐matched healthy controls (HCs; *n* = 172 ranging from underweight to obesity) to exclude BMI as a confound.

**Results:**

Within HCs, higher BMI was associated with higher negative emotional eating and lower happiness eating. AN‐R reported the lowest degree of negative emotional eating relative to other EDs and BMI‐matched HCs, and the highest degree of happiness eating relative to other EDs. The BN and BED groups showed higher negative emotional eating compared to BMI‐matched HCs. Patients with AN‐BP occupied an intermediate position between AN‐R and BN/BED and reported less happiness eating compared to BMI‐matched HCs.

**Discussion:**

Negative emotional and happiness eating patterns differ across EDs. BMI‐independent emotional eating patterns distinguish ED subgroups and might be related to the occurrence of binge eating versus restriction. Hence, different types of emotional eating can represent fruitful targets for tailored psychotherapeutic interventions. While BN and BED might be treated with similar approaches, AN‐BP and AN‐R would need specific treatment modules.

## Graphical abstract

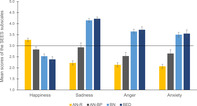

## 1 INTRODUCTION

### 1.1 Eating disorders and their relation with body mass

Three main eating disorders (ED), namely anorexia nervosa (AN), further separable into a restrictive (AN‐R) and a binge‐purge (AN‐BP) subtype, bulimia nervosa (BN) as well as binge‐eating disorder (BED) can be distinguished according to the fifth version of the Diagnostic and Statistical Manual of Mental Disorders (DSM‐5; American Psychiatric Association, 2013). Severe underweight due to energy intake restriction, is a defining feature of AN but also a physical comorbidity that likely influences the more psychological diagnostic symptoms (e.g., body image disturbance). Although the maintenance of self‐starvation behaviors in AN is currently poorly understood, theoretical models emphasize the relationship with emotions (Haynos & Fruzzetti, 2011). Similarly, binge eating (i.e., eating an unusual large amount of food and experiencing a loss of control) which characterizes BN and BED, is thought to be maintained by its effects on mood and emotions (e.g., De Young et al., 2013; De Young, Zander, & Anderson, 2014). Possibly as a result of binge eating, BN and particularly BED are associated with elevated body mass index (BMI)(Hudson, Hiripi, Pope Jr, & Kessler, 2007). Thus, the relationship of disordered eating behaviors like binge eating vs. restriction on the one hand and emotions on the other seem to play a key role for symptom presentation among the EDs. Moreover, BMI and EDs seem to be intrinsically linked, raising the question whether some of the disordered eating behaviors in ED patients might be related to their body weight.

### 1.2 Emotional eating as a correlate of dysfunctional eating behavior in ED psychopathology

Eating in response to negative emotions is important for “hedonic eating” (Burgess, Turan, Lokken, Morse, & Boggiano, 2014; Verhoeven, Adriaanse, de Vet, Fennis, & de Ridder, 2015), a dysfunctional uncoupling of food intake from homeostatic processes like hunger and satiety. Apart from negative emotions, also positive emotions may impact eating behavior, so that research started to acknowledge the role of positive emotions for increased food intake (e.g., Bongers, Jansen, Havermans, Roefs, & Nederkoorn, 2013a; Evers, Dingemans, Junghans, & Boevé, 2018). A link between negative emotions and overeating or binge eating has been documented in several weight‐related groups and EDs across psychometric (e.g., Nicholls, Devonport, & Blake, 2016), experimental (e.g., Cardi, Leppanen, & Treasure, 2015b; Nicholls et al., 2016) and ambulatory research (e.g., Haedt‐Matt & Keel, 2011; Nicholls et al., 2016). Theoretically, overeating or binge eating can be seen as a maladaptive way to cope with negative emotions (Heatherton & Baumeister, 1991) and thus may contribute to the maintenance of ED. In contrast, the role of positive emotions for overeating and binge eating in clinical groups including BN and BED is inconclusive (Braden, Musher‐Eizenman, Watford, & Emley, 2018; Cardi, Esposito, Clarke, Schifano, & Treasure, 2015a; Nicholls et al., 2016), and research indicates that negative compared to positive emotional eating might be more strongly associated with binge eating in non‐clinical samples (Sultson, Kukk, & Akkermann, 2017).

In contrast to binge eating/overeating, links of emotional eating with restricted eating and self‐starvation are less well understood. Several authors have argued that anorectic self‐starvation may serve to facilitate avoidance of negative emotions (Schmidt & Treasure, 2006). Consistent with this idea, individuals with AN showed increased symptoms after negative mood induction (Wildes, Marcus, Bright, & Dapelo, 2012), reported that restrictive eating helps them managing their negative emotions (Espeset, Gulliksen, Nordbø, Skårderud, & Holte, 2012; Nordbø, Espeset, Gulliksen, Skårderud, & Holte, 2006), and exhibited greater dietary restriction subsequent to days marked by negative affect in daily life (Engel et al., 2013). This suggests that patients with AN also resort to altered eating to deal with upsetting emotions, just that they *decrease* food intake instead of the *increase* as seen in binge eating. In contrast, positive emotions compared to a neutral state have been shown to be associated with greater food consumption in AN in a laboratory task (Cardi, Esposito, et al., 2015a). Thus, it might be useful to look into how emotions affect food intake (increase, decrease) in EDs, to understand differences in the clinical presentation of various subtypes of EDs.

Thus far, despite the high relevance of potential maintaining factors of self‐starvation (in AN) and binge eating (in BN and BED) and the obvious role of emotions in this, research investigating emotional eating across various EDs is surprisingly scarce. Moreover, the contribution of mere over−/underweight to emotional eating of the different ED subgroups has not been sufficiently addressed thus far, despite clear evidence that BMI and emotional eating are correlated (e.g., Varela, Andrés, & Saldaña, 2020) and BMI thus represents a potential confound in any comparison between ED groups and HCs. Previous research quite consistently shows that patients with BED score higher on negative emotional eating scales compared to weight‐matched controls (Escandón‐Nagel, Peró, Grau, Soriano, & Feixas, 2018; Pinaquy, Chabrol, Simon, Louvet, & Barbe, 2003; Schulz & Laessle, 2010). Similarly, AN‐BP and BN groups mainly exhibited higher emotional eating scores than non‐weight‐matched HCs (Fioravanti et al., 2014; Ricca et al., 2012; Wardle, 1987), but also no significant differences between AN‐BP and non‐weight‐matched HCs have been found (Baños et al., 2014). However, compared to non‐weight‐matched HCs, individuals with AN‐R exhibited lower (e.g., Danner, Evers, Stok, van Elburg, & de Ridder, 2012), higher (e.g., Ricca et al., 2012), or comparable emotional eating (Baños et al., 2014; Fioravanti et al., 2014). Furthermore, patients with BN scored higher on emotional eating compared to individuals with overweight, (Wardle, 1987). Even subtypes of AN might show specific emotional eating patterns: individuals with AN‐BP exhibited higher emotional eating scores than individuals with AN‐R (Kiezebrink, Campbell, Mann, & Blundell, 2009; Vervaet, van Heeringen, & Audenaert, 2004), potentially attributable to the occurrence of binge eating in the AN‐BP group. Another psychometric study showed that individuals with AN‐R reported lower negative emotional eating scores compared to a binge‐purge group consisting of AN‐BP and BN (Danner et al., 2012). However, Ricca et al. (2012) found no significant differences in emotional eating scores between AN‐R, AN‐BP and BN. Baños et al. (2014) showed higher emotional eating scores in individuals with obesity compared to AN. Thus, there seem to be clear differences in emotional eating across different EDs and weight‐related groups. Yet, previous research has not studied emotional eating, within one study, in individuals with various ED diagnoses while also considering the potential role of BMI in emotional eating.

An examination of emotional eating (both in response to negative and positive emotions) requires a measurement instrument that taps into both “directions” of eating changes, that is, under‐ and overeating. We have recently developed the Salzburg Emotional Eating Scale (SEES) that differentiates between increased eating (i.e., a potential correlate for binge eating and overeating) and decreased eating (i.e., a potential correlate for self‐starvation) in response to emotions (Meule, Reichenberger, & Blechert, 2018). It further differentiates the negative emotions sadness, anxiety and anger and includes a subscale for positive emotions as previous research showed that specific emotions might differ in their impact on eating behavior (e.g., Braden et al., 2018; Macht, 2008). Healthy individuals indeed revealed characteristic emotional *over*eating patterns in response to sadness, *under*eating in response to anxiety and anger and *unchanged* eating behavior in response to happiness, with negative emotional eating subscales and happiness showing divergent correlations with dimensional ED symptom measures (Meule et al., 2018). In a pre‐analysis of 42% of the current sample (see also Supplement A), we examined the SEES scores (among measures of depression, eating psychopathology and emotion regulation) of patients with AN‐R and BN, showing that patients with AN‐R decreased their food intake in response to negative but increased their food intake in response to positive emotions compared to HCs and BN, who showed the opposite pattern (Meule et al., 2019). Yet, the role of positive emotions (compared to negative emotions) on eating behavior is generally less clear (Evers et al., 2018; Nicholls et al., 2016) with only few studies investigating positive emotional eating, especially among samples with EDs or overweight and obesity.

The current study thus follows up on this research and investigates the shared and unique characteristics of emotional eating across all major EDs (AN‐R AN‐BP, BN, and BED), based on interview‐based diagnostics. In addition, we considered the role of BMI by also investigating weight‐related groups (i.e., overweight and obesity) and by selecting BMI‐matched healthy control groups for each ED subgroup. We separately examined the differences between ED groups on the four emotional eating subscales (happiness, sadness, anger and anxiety eating) of the SEES. Based on previous research, we hypothesized higher *negative emotional eating* scores in the BN and BED groups compared to the AN‐R group (Baños et al., 2014; Meule et al., 2019; Wardle, 1987), but because of a lack of previous literature, we had no hypotheses as to whether BN and BED groups differ from each other. Although previous research showed that in BN, binge eating was associated with anger, whereas in BED it was related to depression (Castellini et al., 2012), which would suggest differences between both groups on specific SEES subscales, one could also speculate that the typically higher BMI in the BED group may result in higher negative emotional scores. Regarding *positive emotional eating*, we expected higher scores in AN‐R, and lower scores in BN. Regarding BMI, higher negative emotional eating and lower positive emotional eating in healthy (i.e., with regard to EDs) individuals with elevated BMI was expected based on previous research (Geliebter & Aversa, 2003; Meule et al., 2018; Nolan, Halperin, & Geliebter, 2010). As we aimed at disentangling the effect of ED symptomatology (e.g., binge eating, restriction) from BMI differences, we compared each ED subgroup with BMI‐matched control groups.

## 2 METHODS

### 2.1 Participants

In total, 376 female participants were included in the present study, which was part of a larger project: healthy controls with regard to EDs (HCs; with underweight *n* = 28, normal weight *n* = 84, overweight *n* = 29, and obesity *n* = 31), patients with restrictive subtype AN (AN‐R; *n* = 64), binge‐purge subtype AN (AN‐BP; *n* = 33), BN (*n* = 71), and BED (*n* = 36). All groups were partially recruited at the University of Salzburg, Austria (mostly HCs) as well as before and during inpatient treatment at the Schoen Clinic Roseneck, Germany (mostly ED groups). All participants were tested with a structured clinical interview (Saß, Wittchen, Zaudig, & Houben, 2003) and the second version of the Eating Disorder Examination (Hilbert & Tuschen‐Caffier, 2016) to confirm or exclude an ED diagnosis. Individuals in the ED groups met the respective DSM‐5 criteria, while individuals in the HC groups were classified according to BMI ranges (e.g., for underweight < 18.5 kg/m^2^, normal weight 18.5–24.99 kg/m^2^, overweight 25.00–29.99 kg/m^2^ and obesity ≥ 30.00 kg/m^2^) and were examined using continuous BMI scores. Exclusion criteria for HCs were a current or lifetime ED as revealed with the abovementioned interview. As a result, 44 individuals from originally 420 individuals who were screened for study participation were excluded because of several reasons (*n* = 16 HCs because of lifetime or subclinical EDs, *n* = 24 subclinical ED patients, *n* = 2 because of missing data on the SEES, *n* = 2 because of study withdrawal after the screening).

ED groups differed with regard to age (*F*_[3,200]_ = 12.9, *p* < .001, partial *η* ^2^ = .163; see also Table 1) and BMI (*F*_[3,200]_ = 160.6, *p* < .001, partial *η* ^2^ = .707), but not with regard to years of education (*F*_[3,200]_ = 1.42, *p* = .239, partial *η* ^2^ = .021).

**TABLE 1:** Descriptive data with regard to body mass index, age, and years of education

	Healthy controls (HCs)								Eating disorders (EDs)							
	Underweight		Normal weight		Overweight		Obesity		AN‐R		AN‐BP		BN		BED	
	*n*	*M* (*SD*)	*n*	*M* (*SD*)	*n*	*M* (*SD*)	*n*	*M* (*SD*)	*n*	*M* (*SD*)	*n*	*M* (*SD*)	*n*	*M* (*SD*)	*n*	*M* (*SD*)
Body mass index (kg/m^2^)*	28	17.6 (.57)	84	21.7 (1.76)	29	26.9 (1.56)	31	37.3 (5.83)	64	15.7 (1.75)	33	16.2 (1.61)	71	23.1 (3.60)	36	31.8 (7.05)
Age (years)*	28	24.0 (4.98)	84	23.1 (6.72)	29	27.2 (8.35)	31	32.7 (9.06)	64	24.3 (10.2)	33	25.6 (9.22)	71	26.3 (9.02)	36	36.4 (10.9)
Years of education	28	15.2 (3.05)	83	14.6 (2.48)	29	15.6 (2.86)	30	14.0 (3.10)	64	14.0 (4.31)	33	14.3 (3.16)	71	14.2 (3.48)	36	15.6 (4.81)
Nationality																
German		13 (46%)		36 (43%)		9 (31%)		8 (26%)		59 (92%)		31 (94%)		61 (86%)		15 (42%)
Austrian		13 (46%)		44 (52%)		16 (55%)		23 (74%)		4 (6%)		1 (3%)		8 (11%)		20 (56%)
Others		2 (7%)		4 (5%)		4 (14%)		—		1 (2%)		1 (3%)		2 (3%)		1 (3%)

* Eating disorder groups significantly differed with regard to these variables.

### 2.2 Measures

#### 2.2.1 Salzburg emotional eating scale (SEES)

The SEES (Meule et al., 2018) is a self‐report measure that assesses the extent to which individuals *perceive* their food intake to be altered in response to emotional experiences. The scale consists of 20 items with answers ranging from 1 (*= I eat much less than usual*) to 5 (*= I eat much more than usual*) with a score of 3 indicating unchanged food intake. The scale differentiates between four emotional states, namely happiness (e.g., “When I am optimistic, …”), sadness (e.g., “When I am depressed, …”), anger (e.g., “When I am irritated, …) and anxiety (e.g., “When I am worried, …), derived from previous factor analyses (Meule et al., 2018). Internal consistency in the present study was Cronbach's *α* = .932 for happiness, *α* = .900 for sadness, *α* = .924 for anger, and *α* = .877 for anxiety.

### 2.3 Procedure

In general, the data of the current study were part of a larger project including psychometric, experimental and naturalistic measures. Data were collected in different waves with slightly different study announcement (see also Supplement B) and different exclusion criteria for the project parts (e.g., left‐handed for the laboratory part; not owning a smartphone for the naturalistic part). Not all participants took part in all of the project parts. Laboratory and naturalistic parts of the project are not of importance for the current study and have previously been published (see also Supplement A; Georgii et al., 2020; Georgii, Schulte‐Mecklenbeck, Richard, Van Dyck, & Blechert, 2020; Reichenberger, Pannicke, Arend, Petrowski, & Blechert, 2020; Richard et al., 2019; Schnepper et al., 2020).

Of importance for the current study, participants were either recruited at the University of Salzburg, with the study being advertised via social media groups, university mailing lists, newspaper articles and word of mouth, or at the Schoen Clinic Roseneck, Germany, either before by email announcement, or during their inpatient stay by flyers and personal announcements in treatment groups. The administration of the SEES differed between both assessment sites due to logistic reasons: At the University of Salzburg, participants completed the SEES, among other questionnaires via online survey platform, subsequent to the diagnostic interview and the naturalistic part. At the Schoen Clinic Roseneck, inpatient participants received paper‐pencil version of the SEES, among other questionnaires, after the diagnostic interview and returned them at the appointment for the laboratory part. All participants signed an informed consent form approved by the local ethics committee of the University of Salzburg, Austria as well as the medical review board of the University of Munich, Germany.

### 2.4 Data analysis

ED groups were compared regarding SEES scores with univariate analysis of variance as well as nonparametric Kruskal–Wallis tests as either normality of dependent variables within groups or variance homogeneity was violated. As we were particularly interested in differences between ED groups, significant group differences were followed up with Scheffé or Games‐Howell (in case of variance homogeneity violations) post‐hoc tests. To test the effect of BMI on SEES scores we calculated bivariate non‐parametric correlations within the HCs including individuals with underweight, normal weight, overweight and obesity. To differentiate the effect of BMI from ED psychopathology on emotional eating, auxiliary analyses provided BMI‐matched HCs for each ED group: All patients of the BN, respectively BED group, were matched with BMI‐similar HCs. In contrast, as both AN groups are defined by BMI and also showed BMIs in the life threatening range, we opted for a different approach: only for the auxiliary analyses were BMI criteria set to ≥16.00 kg/m^2^ to derive mild and moderate subsamples of AN‐R and AN‐BP and we compared those to underweight HCs. Effect sizes are reported in terms of partial *η* ^2^ and Hedges'g in case of significant findings. 95% confidence intervals are reported and the alpha level was set at *p* < .05. Data of the current study are available upon request.

## 3 RESULTS

Descriptive data from the groups (i.e., HCs and four ED groups) can be seen in Table 1.

### 3.1 SEES—Happiness

**FIGURE 1:**
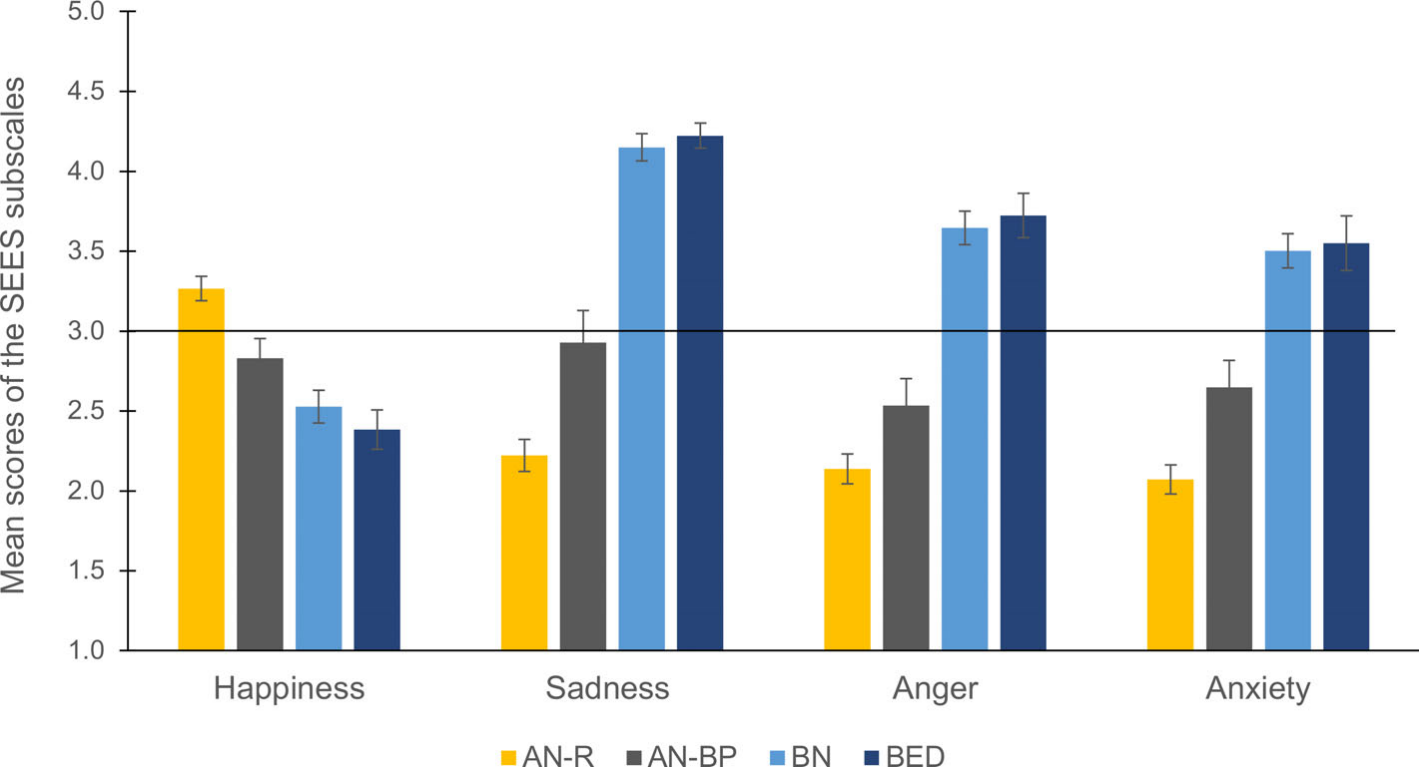
Mean scores of the Salzburg Emotional Eating Scale (SEES) separately for eating disorder groups. AN‐R, Anorexia Nervosa, restrictive subtype; AN‐BP, Anorexia Nervosa, binge‐purge subtype; BN, Bulimia Nervosa; BED, Binge‐Eating Disorder. The scale ranges from “eating much less than usual” (=1) to “eating much more than usual” (=5) with 3 marking the middle point of “eating as much as usual” (=3); solid line. Error bars indicate one standard error of the mean [Color figure can be viewed at wileyonlinelibrary.com]

ED patient groups differed significantly on the SEES happiness eating scores (*F*_(3,200)_ = 15.3, *p* < .001, partial *η* ^2^ = .186 [.090; .271] or nonparametric: *χ* ^2^_(3)_ = 44.9, *p* < .001). As can be seen in Figure 1 (note: an auxiliary Figure 1 is provided in Supplement C, including HCs in the normal BMI range for visual comparisons), happiness eating scores were lowest in BED and increased to BN, AN‐BP, up to AN‐R. Games‐Howell post‐hoc analysis revealed significant differences between AN‐R and all other ED groups, namely AN‐BP (.435 [.052; .818], *p* = .020), BN (.739 [.405; 1.07], *p* < .001) and BED (.882 [.502; 1.26], *p* < .001).

### 3.2 SEES—Sadness

ED patient groups also differed significantly on the SEES sadness eating scores (*F*_(3,200)_ = 83.1, *p* < .001, partial *η* ^2^ = .555 [.461; .619] or nonparametric: *χ* ^2^_(3)_ = 105.7, *p* < .001). Sadness eating scores were lowest in AN‐R and increased to AN‐BP, BN, up to BED (see Figure 1). Games‐Howell post‐hoc analysis revealed significant differences between AN‐R and all other ED groups, namely AN‐BP (−.705 [−1.31; −.106], *p* = .015), BN (−1.93 [−2.27; −1.58], *p* < .001) and BED (−2.00 [−2.34; −1.67], *p* < .001). Additionally, AN‐BP significantly differed from BN (−1.22 [−1.81; −.637], *p* < .001) and BED (−1.30 [−1.87; −.716], *p* < .001).

### 3.3 SEES—Anger

ED patient groups further differed significantly on the SEES anger eating scores (*F*_(3,200)_ = 47.8, *p* < .001, partial *η* ^2^ = .418 [.311; .496] or nonparametric: *χ* ^2^_(3)_ = 88.9, *p* < .001). Anger eating scores were lowest in AN‐R and increased to AN‐BP, BN, up to BED (see Figure 1). Scheffé post‐hoc analysis revealed significant differences between AN‐R and BN (−1.51 [−1.92; −1.10], *p* < .001) and BED (−1.59 [−2.08; −1.09], *p* < .001). Additionally, AN‐BP significantly differed from BN (−1.11 [−1.62; −.608], *p* < .001) and BED (−1.19 [−1.77; −.613], *p* < .001).

### 3.4 SEES—Anxiety

ED patient groups differed significantly on the SEES anxiety eating scores (*F*_(3,200)_ = 36.9, *p* < .001, partial *η* ^2^ = .356 [.247; .439] or nonparametric: *χ* ^2^_(3)_ = 75.2, *p* < .001). Anxiety eating scores were lowest in AN‐R and increased to AN‐BP, BN, up to BED (see Figure 1). Games‐Howell post‐hoc analysis revealed significant differences between AN‐R and all other ED groups, namely AN‐BP (−.577 [−1.08; −.069], *p* = .020), BN (−1.43 [−1.80; −1.06], *p* < .001) and BED (−1.48 [−1.99; −.964], *p* < .001). Additionally, AN‐BP significantly differed from BN (−.853 [−1.38; −.327], *p* < .001) and BED (−.902 [−1.53; −.270], *p* = .002).

### 3.5 Analysis of the association of BMI and SEES scores in healthy individuals

**FIGURE 2:**
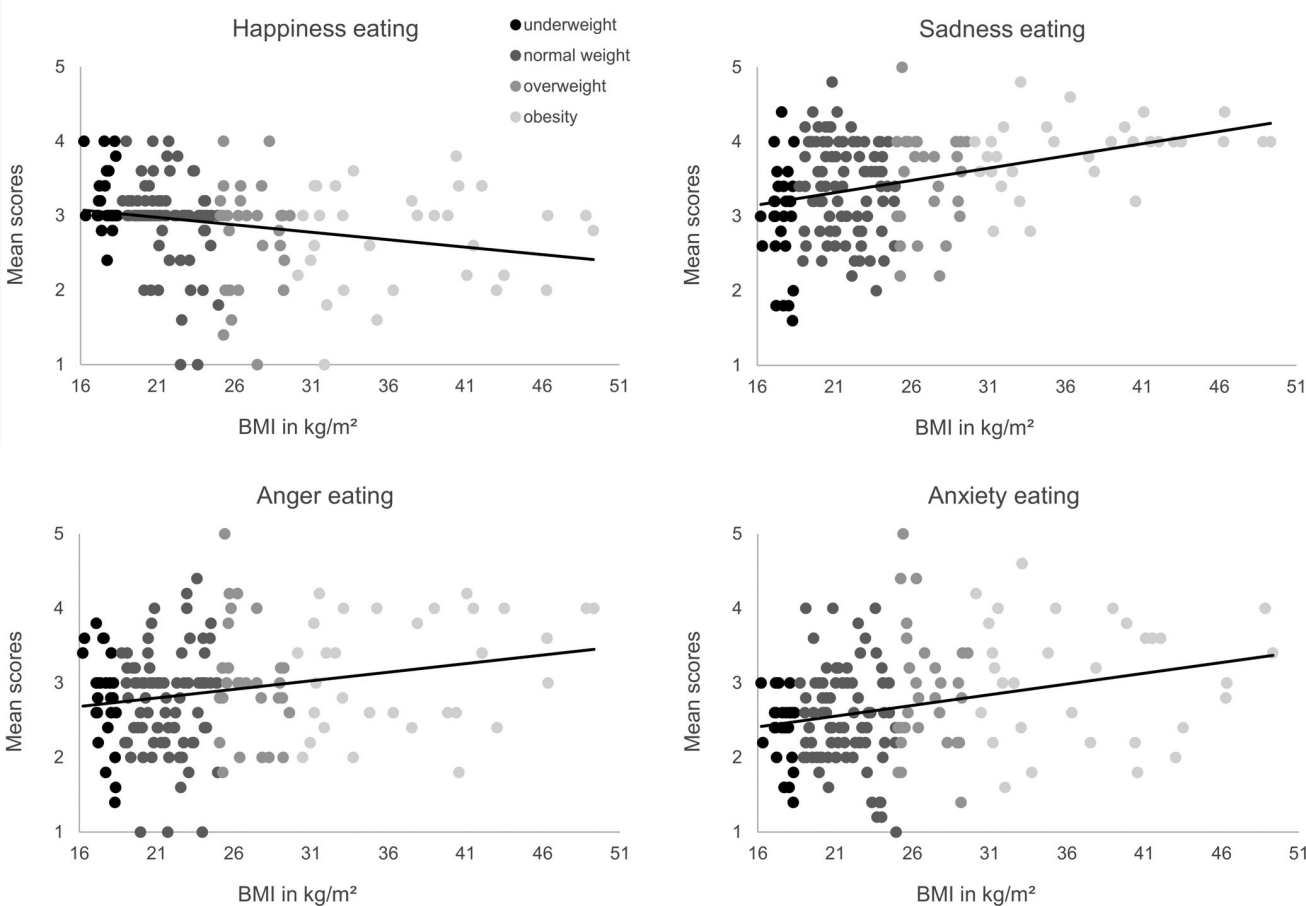
Spearman's rank correlation of the subscales of the Salzburg Emotional Eating Scale with body mass index (BMI) in healthy individuals including individuals with underweight, normal weight, overweight and obesity

In HCs higher BMI correlated negatively with happiness eating, *r*(172) = −.294, *p* < .001 and positively with sadness eating, *r*(172) = .322, *p* < .001, anger eating, *r*(172) = .161, *p* = .034, and anxiety eating, *r*(172) = .264, *p* < .001 (Figure 2).

### 3.6 AN—Auxiliary analyses of emotional eating with BMI‐matched healthy control group

As BMI confounds the above reported comparisons of ED groups, each of the ED groups was additionally compared to weight‐matched HCs. Only individuals with mild and moderate AN were used in the auxiliary analyses. In these subgroups, BMI did not differ significantly between HC_ANmm_matched_ and AN‐R_mm_ or AN‐BP_mm_ (see Table 2).

**TABLE 2:** Descriptive body mass index (BMI) data for auxiliary analyses of eating disorders (EDs) with their BMI‐matched healthy controls (HCs)

	HCs		EDs			
	*n*	*M*(*SD*)	*n*	*M*(*SD*)	Test statistic	*p*
BMI – HC_ANmm_matched_ vs. AN‐R_mm_	28	17.6 (0.57)	31	17.3 (0.67)	*t*_(57)_ = −1.98	.053
BMI – HC_ANmm_matched_ vs. AN‐BP_mm_	28	17.6 (0.57)	18	17.4 (0.81)	*t*_(44)_ = −.916	.365
BMI – HC_BN_matched_ vs. BN	71	23.2 (3.57)	71	23.1 (3.60)	*t*_(140)_ = .104	.971
BMI – HC_BED_matched_ vs. BED	36	30.2 (7.73)	36	31.8 (7.05)	*t*_(70)_ = .910	.366

Abbreviations: AN‐R_mm_, mild/moderate subgroup of restrictive anorexia nervosa; AN‐BP_mm_, mild/moderate subgroup of binge‐purge anorexia nervosa; BN, bulimia nervosa; BED, binge‐eating disorder; BMI‐matched healthy individuals (HC_ANmm_matched_, HC_BN_matched_, HC_BED_matched_).

**FIGURE 3:**
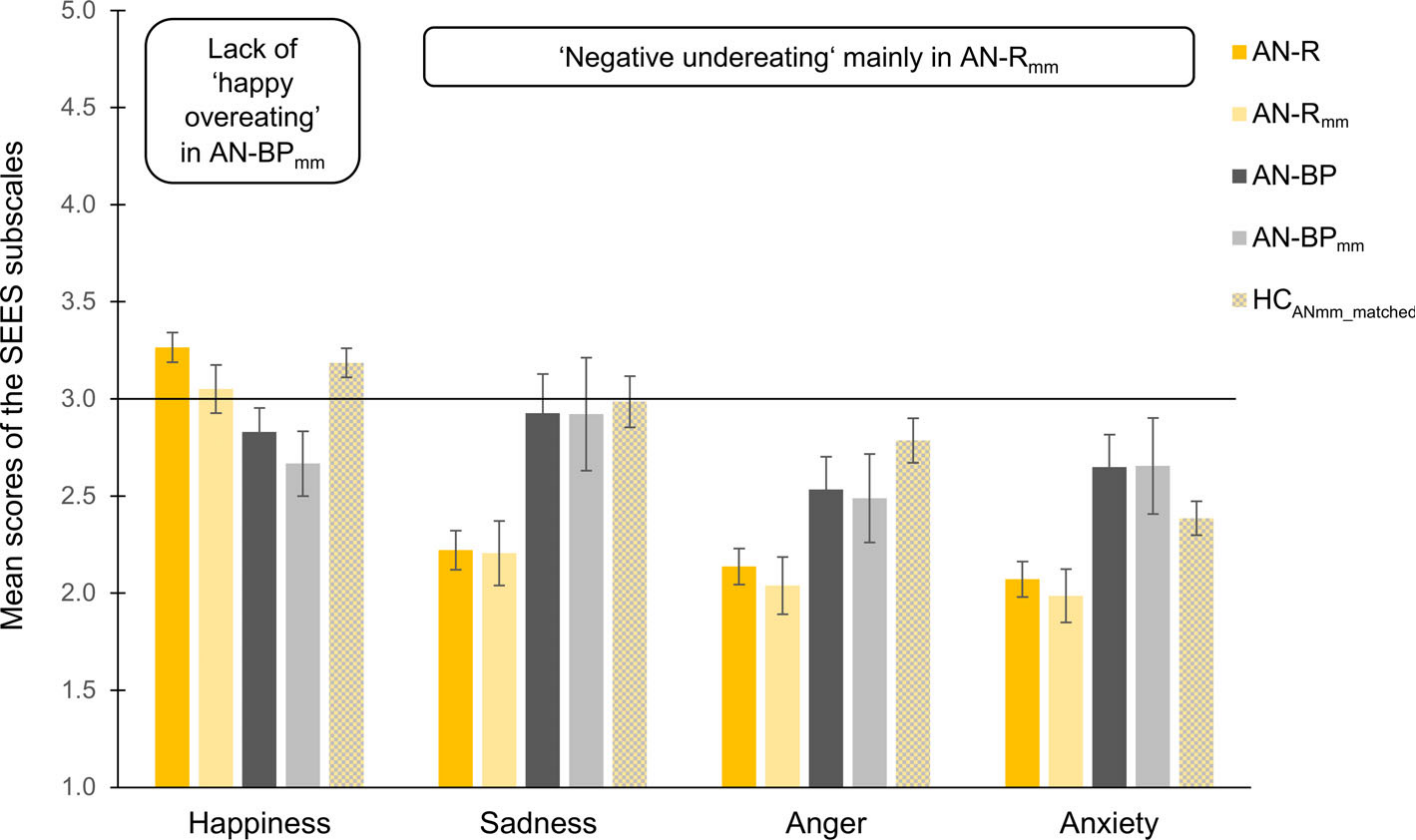
Mean scores of the Salzburg Emotional Eating Scale (SEES) as a function of the whole restrictive‐subtype Anorexia Nervosa (AN‐R) group and the mild/moderate subgroup (AN‐R_mm_), the whole binge‐purge‐subtype Anorexia Nervosa (AN‐BP) group and the mild/moderate subgroup (AN‐BP_mm_), and BMI‐matched healthy individuals HC_ANmm_matched_. The scale ranges from “eating much less than usual” (=1) to “eating much more than usual” (=5) with 3 marking the middle point of “eating as much as usual” (=3); solid line. Error bars indicate one standard error of the mean [Color figure can be viewed at wileyonlinelibrary.com]

As can be seen in Figure 3 and Table 3, whereas AN‐R_mm_ and HC_ANmm_matched_ did not differ with regard to happiness eating, AN‐BP_mm_ exhibited lower happiness eating compared to HC_ANmm_matched_. AN‐R_mm_ patients showed lower sadness, anxiety and anger eating, but AN‐BP_mm_ exhibited comparable levels of sadness, anxiety and anger eating compared to HC_ANmm_matched_.

**TABLE 3:** Comparisons of eating disorder groups with their respective weight‐matched controls

	Statistic	*p*‐Value	CI 95%	Effect size (Hedges'g)
AN‐R_mm_ versus HC_ANmm_matched_				
Happiness eating	*t*_(57)_ = −.902	.371		
Sadness eating	*t*_(57)_ = −3.62	.001	[−1.21; −.348]	.932
Anxiety eating	*t*_(57)_ = −2.40	.020	[−.732; −.066]	.617
Anger eating	*t*_(57)_ = −3.96	<.001	[−1.12; −.369]	1.02
AN‐BP_mm_ versus HC_ANmm_matched_				
Happiness eating	*t*_(23.95)_ = −2.84	.009	[−.896; −.142]	.949
Sadness eating	*t*_(44)_ = −.223	.824		
Anxiety eating	*t*_(21.29)_ = 1.03	.315		
Anger eating	*t*_(25.55)_ = −1.16	.255		
BN versus HC_BN_matched_				
Happiness eating	*t*_(125.6)_ = .515	.608		
Sadness eating	*t*_(140)_ = −5.65	<.001	[−.867; −.417]	.942
Anxiety eating	*t*_(140)_ = −6.05	<.001	[−1.11; −.561]	1.01
Anger eating	*t*_(133.9)_ = −6.18	<.001	[−1.10; −.565]	1.03
BED versus HC_BED_matched_				
Happiness eating	*t*_(70)_ = 2.83	.006	[.133; .767]	.659
Sadness eating	*t*_(70)_ = −4.94	<.001	[−.928; −.394]	1.15
Anxiety eating	*t*_(70)_ = −3.98	<.001	[−1.31; −.434]	.927
Anger eating	*t*_(70)_ = −4.54	<.001	[−1.13; −.440]	1.06

Abbreviations: AN‐R_mm_, mild/moderate subgroup of restrictive anorexia nervosa; AN‐BP_mm_, mild/moderate subgroup of binge‐purge anorexia nervosa; BN, bulimia nervosa; BED, binge‐eating disorder; BMI‐matched healthy individuals (HC_ANmm_matched_, HC_BN_matched_, HC_BED_matched_); CI, confidence interval.

### 3.7 BN and BED—auxiliary analyses of emotional eating with weight‐matched healthy control group

The BN and the BED groups were separately matched with individuals of the HCs group and neither BN and HC_BN_matched_ nor BED and HC_BED_matched_ significantly differed with regard to BMI (Table 2).

**FIGURE 4:**
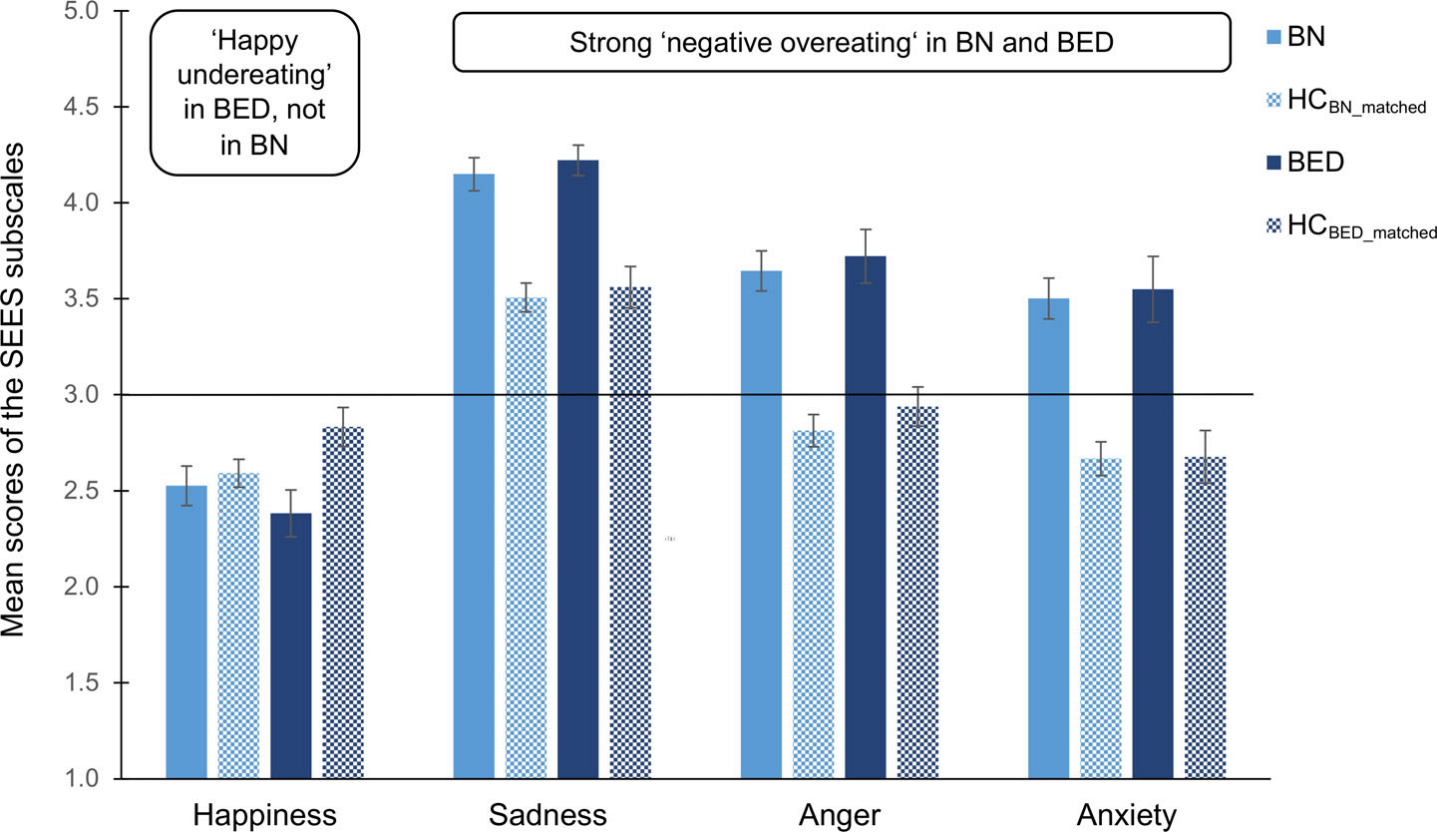
Mean scores of the Salzburg Emotional Eating Scale (SEES) as a function of the Bulimia Nervosa (BN) group, the Binge‐Eating Disorder (BED) group and their respective weight‐matched healthy individuals groups (HC_BN_matched_ and HC_BED_matched_, respectively). The scale ranges from “eating much less than usual” (=1) to “eating much more than usual” (=5) with 3 marking the middle point of “eating as much as usual” (=3); solid line. Error bars indicate one standard error of the mean [Color figure can be viewed at wileyonlinelibrary.com]

As can be seen in Figure 4 and Table 3, compared to the subsample of HC_BN_matched_, BN had comparable levels of happiness eating, whereas the BED group exhibited significantly lower happiness eating scores compared to the subsample of HC_BED_matched_. Both, BN and BED exhibited higher scores on sadness, anxiety and anger eating compared to their respective weight‐matched controls (i.e., HC_BN_matched_ and HC_BED_matched_).

## 4 DISCUSSION

The aims of the present study were to characterize all relevant ED subgroups with regard to their emotional eating patterns (a), to examine whether and how BMI is related to various types of emotional eating (b), and to refine emotional eating by controlling for BMI differences between ED groups (c). The main findings of this study demonstrated significant differences between the EDs with increasing levels of negative emotional eating and decreasing levels of happiness eating from AN‐R, AN‐BP, BN to BED (a). In HCs higher BMI was associated with higher negative emotional eating and lower happiness eating (b). This demonstrated the necessity to control ED subgroup differences in emotional eating for BMI (c): Compared to weight‐matched healthy individuals, only the mild/moderate AN‐R group reported lower negative emotional eating (“negative undereating”) while the BN and the BED groups reported clearly higher negative emotional eating (“negative overeating”). The AN‐BP group reported lack of “happy overeating,” while the BED group further reported “happy undereating.”

### 4.1 Differences between ED groups with regard to negative emotional eating

Negative emotional eating was low in AN‐R and gradually increased in AN‐BP, BN to BED. Across groups, sadness seemed to contribute the most to such negative emotional overeating, in line with previous research using a sadness mood induction (van Strien et al., 2013). Whereas AN‐R and AN‐BP differed from BN/BED with regard to all negative emotional eating subscales, AN‐R and AN‐BP differed from each other only on sadness and anxiety eating, whereas BN and BED did not differ from each other on any of the negative emotional eating subscales.

Relative to the midpoint of the scale (“no change in eating,” 3), patients with AN‐R and AN‐BP reported eating *less* when sad, anxious or angry which further suggests that individuals with AN may use self‐starvation as a form of regulation of negative emotions (Brockmeyer et al., 2012; Meule et al., 2019). Notably, patients with AN‐BP reported higher scores on negative emotional eating compared to patients with AN‐R, despite similar BMI. This contrasts with research finding no significant differences between both AN groups with regard to emotional eating (Baños et al., 2014; Ricca et al., 2012). However, it underpins the important role of binge eating in the AN‐BP group only, in line with previous research showing that emotional eating was higher in patient groups characterized by binge eating or purging behavior (e.g., Masheb & Grilo, 2006; Ricca et al., 2009) and lower in groups characterized by restrictive behaviors (e.g., Rotella et al., 2018). Hence, the emotional eating expressions and potentially also the emotion regulation schema might differ between both AN groups: the AN‐R group uses self‐starvation only whereas AN‐BP might rely more on binge eating interleaved with highly restrictive periods to regulate negative emotions (DeJong et al., 2013; Reas & Rø, 2018). Still, AN‐BP exhibited significantly lower negative emotional eating than the BN and BED groups, suggesting a distinct role of AN‐BP at an intermediate position between the other ED groups.

Patients with BN and BED did not differ with regard to negative emotional eating suggesting that the similarities of the two groups with regard to binge eating arise on a shared negative emotional eating background, while the compensation—seen only in BN—does not alter the emotional eating expression. This contrasts with research suggesting that specific emotions might be more important for triggering emotional eating in patients with purging behavior (Rotella et al., 2018) or in patients with BN in contrast to BED (Castellini et al., 2012). However, in general, the BN and BED groups rather report overeating in response to negative emotions, supporting the emotion regulation model of overeating (or in extreme forms binge eating) in BN and BED stating that eating might be used to cope with and alleviate any kind of negative emotion (e.g., Leehr et al., 2015).

### 4.2 Differences between ED groups with regard to positive emotional eating

In line with previous suggestions to include positive emotions into the definition of emotional eating (e.g., Bongers, Jansen, Houben, & Roefs, 2013b; Evers, Adriaanse, de Ridder, & de Witt Huberts, 2013; Meule et al., 2018), our results lend further support for the important role of positive emotional eating, especially in differentiating between ED subgroups and between ED subgroups and weight‐matched HCs. “Happy overeating” was lowest in BED (lower than in weight‐matched HCs), and increased toward BN, AN‐BP to AN‐R. Relative to the scale midpoint, whereas AN‐BP, BN and BED reported decreased, AN‐R reported increased happiness eating. This finding is in line with our pre‐analysis of the data (Meule et al., 2019) and laboratory research showing that induction of positive mood resulted in increased food intake in patients with AN (Cardi, Esposito, et al., 2015a) and suggests that positive mood might facilitate “self‐caring behaviors” in individuals with AN‐R. Interestingly, AN‐BP did not share this pattern of elevated happy eating. Patients with BED reported less frequent overeating in response to happiness compared to other emotions (Masheb & Grilo, 2006). Our results mirror that and suggest that BED patients compensate for their “negative overeating” through “happy undereating,” similar to previous research in stress eating (Sproesser, Schupp, & Renner, 2014).

### 4.3 The relationship of BMI and emotional eating in healthy individuals

In line with previous research (e.g., Meule et al., 2018; Nolan et al., 2010), higher BMI was significantly associated with lower happy (over)eating as well as higher sadness, anxiety and anger eating in our HCs. However, results are in contrast to van Strien, Donker, and Ouwens (2016) who found positive emotional eating being unrelated to BMI in a sample of individuals with overweight or obesity. Again, our results point to some *emotion specificity* in emotional eating: Sadness eating exhibited the strongest positive correlation, with anger and anxiety showing significant but lower, positive correlations. Additionally, the present results further support the notion that at least in HCs, a style of “happy overeating” (or the flip side: “unhappy undereating”) may be more functional with regard to weight management compared to a style of “unhappy overeating” (Meule et al., 2018). Indeed, previous research showed that negative emotional eating might be a risk factor for longitudinal weight gain (Frayn & Knäuper, 2018; van Strien, Herman, & Verheijden, 2012).

### 4.4 The role of BMI and ED psychopathology (i.e., binge eating or self‐starvation) for emotional eating

The present study aimed at disentangling the role of BMI from binge eating for emotional eating. The current findings showed higher negative emotional eating in BN and BED groups relative to their BMI‐matched HCs. This suggests that binge eating has its psychometric expression in negative emotional (over)eating in these groups and emotional eating is not mainly influenced by BMI or the presence of compensatory behavior. By contrast, patients with AN‐R showed less negative emotional eating than their BMI‐matched HCs, suggesting that the symptomatic dietary restriction and not BMI seems to contribute to negative emotional (under)eating in this group. Interestingly, the AN‐BP group seemed most similar to BMI‐matched HCs, as they only differed with regard to happiness eating, potentially because their behavior is marked by binge eating and restrained eating at the same time. Hence, whereas BMI seems to be an important factor for negative emotional eating in healthy individuals, emotional eating in ED groups seems mainly to be related to binge eating on the one hand, restrained eating on the other, and not BMI.

### 4.5 Strengths, limitations, and future research

Several strengths of the present study such as thorough interview diagnostics and mostly large sample sizes alongside with the inclusion of a large control group with a broad BMI range have to been seen in the context of some limitations. First, the present study used a psychometric, cross‐sectional design limiting the causal inference of the interpretation of the results. The SEES is a self‐report questionnaire that measures perceived alterations in food intake in response to emotions. Hence, the scale is susceptible to response and retrospective biases and several researchers argued that emotional eating questionnaires are not validly assessing real behavior (Bongers & Jansen, 2016; Evers, de Ridder, & Adriaanse, 2009). Yet, we have reported findings that support the validity of emotional eating scales with regard to craving and appetitive responding (e.g., Reichenberger et al., 2018; Schnepper et al., 2020). Still, future research should corroborate the present results, for example, through measuring actual food intake in laboratory or ecological momentary assessment studies. Second, although most groups were well powered, the patient group of AN‐BP was relatively small in comparison to the other groups, suggesting caution when generalizing some of the results. Third, groups differed with regard to age and future research might profit from age matched groups. However, it has previously been shown that one characteristic of EDs are different age onsets (e.g., a rather early onset of AN compared to a rather late onset of BED) (Hudson et al., 2007) making age matching a complex issue. Fourth, all studied participants were female, so that the findings cannot be generalized to male individuals. Although females have been shown to exhibit greater ED symptomatology (Lewinsohn, Seeley, Moerk, & Striegel‐Moore, 2002), EDs in male individuals should not be underestimated (Limbers, Cohen, & Gray, 2018; Murray et al., 2017). The present study excluded male participants because of generally higher emotional (binge) eating in females compared to males (Tanofsky, Wilfley, Spurrell, Welch, & Brownell, 1997) and potentially different symptom profiles in males, which conflicted with our aim for homogenous, reasonably sized ED subgroups. Fifth, the participants (especially AN and BN) who completed questionnaires during their in‐patient treatment might have already gained a better understanding of their eating behavior. Future research might circumvent this variability by fixed assessment at the beginning of the in‐patient stay. Fifth, HCs did not exhibit current or lifetime EDs, however, participants might still have fulfilled criteria for current other psychological disorders. However, we refrained from recruiting a sample without any psychological disorders to avoid confounding our results by a “general psychopathology” factor.

### 4.6 Clinical implications and outlook

It has previously been argued that psychotherapeutic treatment should be tailored to the degree of emotional eating: Individuals with high emotional eating might especially profit from interventions improving emotion regulation skills or mindfulness (van Strien, 2018). Indeed, these approaches have shown promising results in reducing emotional eating meanwhile facilitating weight loss (Frayn & Knäuper, 2018). Our results support this and further identify more distinct emotional eating patterns in each ED subgroup by specifying the direction of eating change (over‐ vs. undereating) as well as the valence (positive vs. negative) and nature (sadness, anger, anxiety) of trigger emotions. One‐dimensional (negative) emotional eating scales would have overlooked several of these group differences. Our findings in AN‐R suggest that facilitating positive emotions may help individuals with AN eat more and thus represent an important content for interventions in order to normalize eating behavior and achieve weight restoration. Importantly, intervening on emotional eating in the same way for all EDs is not consistent with the present findings which provide a clear tailoring guideline for subgroup specific interventions: while BN and BED might be treated with similar approaches, AN‐BP and AN‐R would need specific treatment modules.

## CONFLICT OF INTEREST

The authors declare no conflict of interest.

## Supporting information


**Appendix S1**: Supporting information

**Appendix S2**: Supporting information

**Appendix S3**: Supporting information

## Data Availability

Data available upon request.
