# Supplementary material for: Emotional eating across different eating disorders and the role of body mass, restriction, and binge eating
Source: Int J Eat Disord. 2021 Mar 3;54(5):773–84. doi: 10.1002/eat.23477 (PMC8252459; doi:10.1002/eat.23477)
Supplement: Supplementary file 1 — Appendix S1: Supporting information [file EAT-54-773-s003.docx]

**Supplement A**

Table 1 provides an overview on other manuscripts that used parts of the larger project with regard to data or participants.

Table 1. Overlap between the current manuscript and other publications.

| **Paper** | **Data overlap** | **Sample overlap** |
| --- | --- | --- |
| Meule, A., Richard, A., Schnepper, R., Reichenberger, J., Georgii, C., Naab, S., Voderholzer, U., & Blechert, J. (2019). Emotion regulation and emotional eating in anorexia nervosa and bulimia nervosa. *Eating Disorders* | SEES | Total N=158  n=53 HCs normal weight  n=13 HCs overweight  n=2 HCs obesity  n=47 AN-R  n=43 BN |
| Georgii, C., Schulte-Mecklenbeck, M., Richard, A., van Dyck, Z., & Blechert, J. (2020). The dynamics of self-control: within-participant modeling of binary food choices and underlying decision processes as a function of restrained eating. *Psychological Research, 84*(7), 1777-1788. | None, use of laboratory parts | Total N=59  n=46 HCs normal weight  n=7 HCs overweight  n=2 HCs obesity  n=4 HCs underweight |
| Reichenberger, J., Pannicke, B., Arend, A.-K., Petrowski, K., & Blechert, J. (2020). Does stress eat away at you or make you eat? EMA measures of stress predict day to day food craving and perceived food intake as a function of trait stress-eating. *Psychology & Health, 24*, 1-19. | None; use of naturalistic parts | Total N=73  n=57 HCs normal weight  n=6 HCs overweight  n=5 HCs obesity  n=5 HCs underweight |
| Schnepper, R., Georgii, C., Eichin, K., Arend, A.-K., Wilhelm, F. H., Vögele, C., Lutz, A. P. C., van Dyck, Z., & Blechert, J. (2020). Fight, Flight, - Or Grab a Bite! Trait Emotional and Restrained Eating Style Predicts Food Cue Responding Under Negative Emotions. *Frontiers in Behavioral Neuroscience,* 14:91*.* | None; use of laboratory parts | Total N=75  n=59 HCs normal weight  n=8 HCs overweight  n=3 HCs obesity  n=5 HCs underweight |
| Richard, A., Meule, A., Georgii, C., Voderholzer, U., Cuntz, U., Wilhelm, F. H., & Blechert, J. (2019). Associations between interoceptive sensitivity, intuitive eating, and body mass index in patients with anorexia nervosa and normal-weight controls. *European Eating Disorder Review, 27*(5), 571-577. | None; use of laboratory parts | Total N=76  n=39 HCs normal weight  n=29 AN-R  n=8 AN-BP |
| Georgii, C., Eichin, K. N., Richard, A., Schnepper, R., Naab, S., Voderholzer, U., Treasure, J., & Blechert, J. (in revision). I change my mind to get better: Mouse-tracing-based micro-analysis of food choice processes reveals differences between Anorexia Nervosa and Bulimia Nervosa during inpatient treatment. | None; use of laboratory parts | Total N=122  n=48 HCs normal weight  n=10 HCs overweight  n=1 HC obesity  n=28 AN-R  n=8 AN-BP  n=27 BN |

*Note*. SEES = Salzburg Emotional Eating Scale. HCs = Healthy Controls; AN-R = Anorexia Nervosa, restrictive subtype; AN-BP = Anorexia Nervosa, binge-purge subtype; BN = Bulimia Nervosa; BED = Binge-Eating Disorder.
