# Supplementary material for: Emotional eating across different eating disorders and the role of body mass, restriction, and binge eating
Source: Int J Eat Disord. 2021 Mar 3;54(5):773–84. doi: 10.1002/eat.23477 (PMC8252459; doi:10.1002/eat.23477)
Supplement: Supplementary file 2 — Appendix S2: Supporting information [file EAT-54-773-s002.docx]

**Supplement B**

Data collection was conducted in different waves with varying inclusion of study parts and slightly different announcements for recruitment:

| **Wave and sampled groups** | **Study parts** | **Announcement** |
| --- | --- | --- |
| 1 – HCs | psychometric  laboratory  naturalistic | ‘Smartphone-based assessment of eating styles and brain reactions towards food’ |
| 2 – AN & BN in-patients | psychometric  laboratory | ‘How and why emotions influence food intake and food choice’ |
| 3 – HCs (especially overweight / obesity) & BED | psychometric  laboratory  naturalistic | ‘Obesity (or binge eating for BED participants), weight problems and diet relapses? Neuroscientific study seeks participants!’ |
| 4 – AN, BN & BED | psychometric  naturalistic | ‘How and why emotions influence food intake’ |
| 5 – HCs | psychometric  naturalistic | ‘Emotions and eating in daily life’ |
| 6 – HCs (especially underweight) | psychometric | ‘Short study of eating behavior for underweight individuals without ED’ |

*Note.* HCs = Healthy Controls; AN = Anorexia Nervosa; BN = Bulimia Nervosa; BED = Binge-Eating Disorder.
