# Supplementary material for: Emotional eating across different eating disorders and the role of body mass, restriction, and binge eating
Source: Int J Eat Disord. 2021 Mar 3;54(5):773–84. doi: 10.1002/eat.23477 (PMC8252459; doi:10.1002/eat.23477)
Supplement: Supplementary file 3 — Appendix S3: Supporting information [file EAT-54-773-s001.docx]

**Supplement C**

**Auxiliary Figure 1**

*Mean scores of the Salzburg Emotional Eating Scale (SEES) separately for eating disorder groups and healthy controls.*


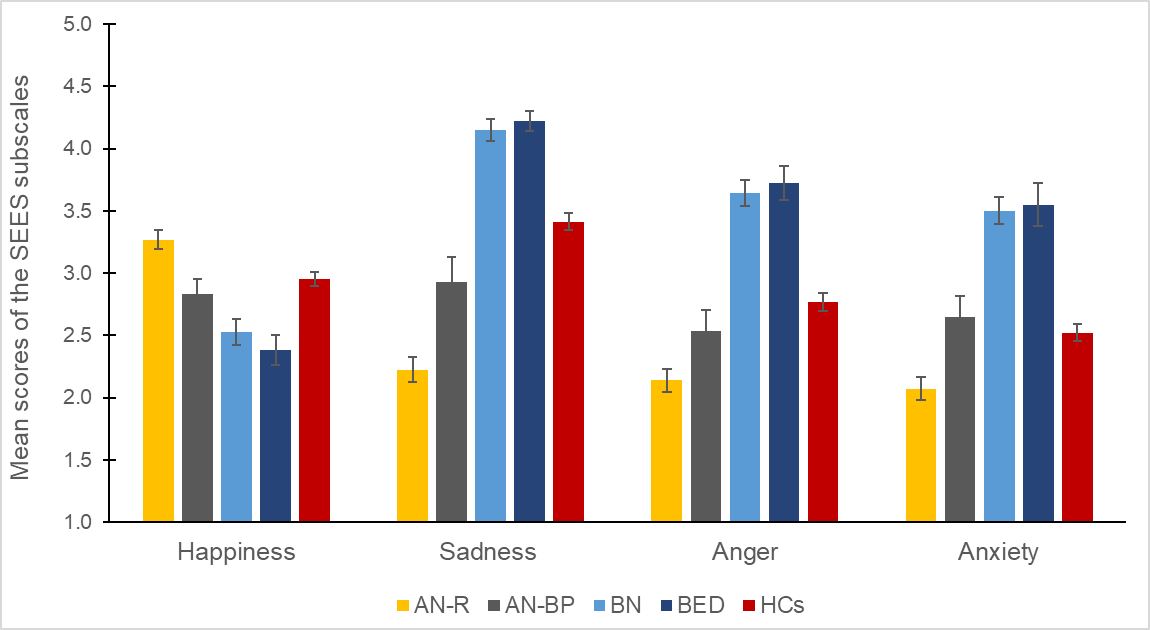


*Note.* AN-R= Anorexia Nervosa, restrictive subtype; AN-BP=Anorexia Nervosa, binge-purge subtype; BN= Bulimia Nervosa; BED= Binge-Eating Disorder; HCs= Healthy Controls with a normal body mass index between 18.5-24.99 kg/m². The scale ranges from ‘eating much less than usual’ (=1) to ‘eating much more than usual’ (=5) with 3 marking the middle point of ‘eating as much as usual’ (=3); solid line. Error bars indicate one standard error of the mean.
